# Primary care providers’ perceptions on the integration of community-led advance care planning activities with primary care: a cross-sectional survey

**DOI:** 10.1186/s12875-023-02144-z

**Published:** 2023-09-24

**Authors:** Rachel Z Carter, Monika Ludwig, Angela Gao, Amy Tan, Doris Barwich, Michelle Howard

**Affiliations:** 1 Department of Medicine, University of British Columbia, 2329 West Mall, Vancouver, BC V6T 1Z4 Canada; 2 British Columbia Centre for Palliative Care, 300-601 Sixth St, New Westminster, BC V3L 3C1 Canada; 3 Michael G. DeGroote School of Medicine, McMaster University, 1280 Main Street West, Hamilton, ON L8S 4L8 Canada; 4 Department of Family Medicine, McMaster University, 1280 Main Street West, Hamilton, ON L8S 4L8 Canada

**Keywords:** Primary health care, Physicians, Advance care planning, Communication, Survey

## Abstract

**Background:**

Advance care planning (ACP) is a process intended to help ensure people receive medical care that is consistent with their values, goals, and preferences during serious and chronic illness. Barriers to implementing ACP in primary care settings exist. Community-led ACP initiatives exist in British Columbia to engage the public directly. These initiatives may help prepare people for conversations with their primary care providers. The objectives of this study were to elicit primary care providers’ perceptions of the utility and desired content of community-led ACP activities and suggestions for integrating community-led ACP activities with primary care.

**Methods:**

We conducted an online cross-sectional survey of primary care providers practicing in British Columbia, Canada in 2021. Both quantitative and qualitative survey questions addressed ACP engagement in practice, the perceived role and desired outcomes of community-led ACP activities, and ways to integrate community-led ACP activities with primary care.

**Results:**

Eighty-one providers responded. Over 80% perceived a moderate or greater potential impact of community-led ACP activities. The most common reasons for not referring a patient to a community-led ACP activity were lack of awareness of the option locally (62.1%) and in general (44.8%). Respondents wanted their patients to reflect on their values, wishes and preferences for care, to have at least thought about their goals of care and to have chosen a substitute decision maker in the community. They indicated a desire for a summary of their patient’s participation and a follow-up discussion with them about their ACP. They suggested ways to integrate referral to programs into existing health care system structures.

**Conclusions:**

Community-led ACP activities were perceived to be useful to engage and prepare patients to continue ACP discussions with clinicians. Efforts should be made to establish and integrate community-based ACP initiatives within existing primary care systems to ensure awareness and uptake.

**Supplementary Information:**

The online version contains supplementary material available at 10.1186/s12875-023-02144-z.

## Background

Advance care planning (ACP) is a process intended to “help ensure people receive medical care that is consistent with their values, goals, and preferences during serious and chronic illness” [1]. Prior engagement in ACP is associated with improved patient and family experiences with healthcare near end of life, greater concordance between patient wishes and the healthcare they receive and fewer unwanted intensive treatments [2, 3]. Patients agree that ACP should be undertaken before a medical crisis, [4–6] and that the topic should be raised by their physician [7, 8]. However, when physicians do initiate these conversations it tends to be late in the illness trajectory [9–11].

Primary care is ideally suited to facilitate aspects of ACP, in part because of the longitudinal nature of the relationships between patients and their primary care providers (PCPs) and the opportunity for ongoing discussions over time [12, 13]. However, PCPs rarely raise the discussion, often citing the perception that patients are not ready and the lack of time in clinical encounters [11, 14–18]. It has been widely recognized that ACP activities such as determining a substitute decision maker (SDM) and discussing values and wishes for healthcare do not need to be initiated in the clinical setting [19].

Many initiatives in Canada and elsewhere have created ACP resources and processes to engage the public directly [20, 21]. For example, the British Columbia Centre for Palliative Care (BCCPC) has supported hundreds of community-based nonprofits across British Columbia (BC) with funding and training to deliver information sessions about ACP to the public [22]. Many, but not all, of the organizations hosting these sessions are Hospice Societies, with sessions facilitated by their staff or volunteers [23]. Organizations typically make use of traditional and social media to market their sessions, as well as through partnerships with other local organizations, such as libraries and community centers. However, community programs for ACP can be siloed from clinical care and are likely to be most impactful when they occur in combination with conversations with a primary care clinician. Community organizations embarking on ACP education programs have reported that lack of public engagement and understanding of ACP are barriers to engaging the public, and better integration between community programs and clinical and health systems are needed [20].

Family physicians have suggested that greater public awareness and engagement in ACP would be beneficial for their efforts to discuss ACP with their patients [16]. There is evidence that use of an online ACP tool by patients can lead to increased reciprocal ACP communication in subsequent visits with the primary care physician [24]. Similarly, community resources and programs exist in BC that could better prepare patients for ACP discussions with their PCPs, however these programs are not currently well integrated with primary care. Knowledge of how best to integrate ACP programs with primary care, including whether and how PCPs would prefer to engage with them could help improve the uptake and effectiveness of ACP.

Therefore, the objectives of this study were to elicit PCP perceptions of the utility and desired content of community-led ACP activities and suggestions for integrating community-led ACP activities with primary care.

## Methods

### Research design and method

A cross-sectional survey was conducted. The target population of this study was primary care providers (PCPs) practicing in British Columbia (BC). To participate, PCPs were required to (1) be a licensed Family Physician/General Practitioner or a Nurse Practitioner, (2) practice in BC, and (3) have sufficient English language skills to complete the survey.

An online survey (see supplementary materials), programmed in Qualtrics, was used to gather data. Survey questions were designed to assess ACP engagement within their primary care practices, the perceived role and desired outcomes of community-led ACP activities, and to identify strategies to integrate community-led ACP activities with primary care. The survey questions were grouped into three sections; (1) ACP in Primary Care (7 Likert-scale, 1 multiple-choice with the option to add choice, 1 free-text); (2) Facilitating ACP through community-based approaches (1 yes/no with prompt to specify information based on answer, 1 yes/no with multiple-choice question based on answer, 2 Likert-scale, 1 with multiple choice or free-text question based on answer, 1 free-text); and (3) Demographics (9 questions, all multiple-choice). Previous studies and reports informed options provided for barriers and facilitators [16, 20, 25]. The survey was reviewed by the research team, which includes a family physician, palliative care physician, and researchers with extensive experience in the area.

### Recruitment & data collection

Data collection took place from January 18 to February 23, 2021. Using a convenience sampling approach, PCPs were recruited by invitation email sent by the Divisions of Family Practice throughout BC to their members (twice, with the second invite sent two weeks after the first) or other communication (e.g., the BCCPC email newsletter).

Reimbursement for the time spent participating in the survey was offered to the first 150 respondents in the form of a $15 electronic gift card. Required contact information (email address) to receive the electronic gift voucher was collected using a separate online survey.

Ethical approval for this study was granted through the University of British Columbia’s Research Ethics Board prior to data collection (H20-03993). Consent to participate was indicated by completion of the survey.

### Data analysis

Information on respondent characteristics and the multiple-choice survey questions were analyzed by calculating percentages of responses in the categories. Responses to open-text questions were analyzed by inductive content analysis [26]. Two authors (AG, ML) independently read the comments, generated codes, and grouped codes into categories, before meeting to review and discuss to achieve consensus. There were no instances where consensus was not reached through discussion. After consensus was reached, categories were abstracted into main categories, and data was re-categorized as required to align with updated categories.

This paper follows the STROBE reporting guidelines [27].

## Results

### Participants

A total of n = 122 entries were recorded during the data collection period. Any entries with computer-generated responses (n = 18) or without any data/responses provided (n = 13) were excluded from further analysis. Respondents that completed the survey in an unrealistically short time period (less than 200 s; n = 3) or did not identify as a PCP (n = 1) were also omitted. Therefore, n = 87 surveys were analyzed. Of those, 6 respondents dropped out in the first quarter of the survey during the background questions on PCP engagement with ACP. Their data was included in the analysis despite being incomplete. No further participants were lost to drop out.

Of the 81 respondents that provided demographic information, the majority identified as female (69%; 56/81), between 35 and 44 years old (34.6%; 28/81), a family physician/general practitioner (91.4%; 74/81) and had provided primary care for less than 5 (30.9%; 25/81) or more than 20 years (30.9%; 25/81) (Table 1). Approximately half (53.1%; 43/81) reported having had additional education, training or certification in end-of-life care, palliative care, or serious illness conversations.

**Table 1:** Profile of survey respondents (n = 81)

Characteristic		N (%)
Gender	Female	56 (69.1%)
	Male	22 (27.2%)
	Prefer not to disclose	3 (3.7%)
Age group	< 35 years	14 (17.3%)
	35–44 years	28 (34.6%)
	45–54 years	18 (22.2%)
	55–64 years	19 (23.5%)
	65–74 years	2 (2.5%)
Type of primary care provider	Family physician/general practitioner	74 (91.4%)
	Nurse practitioner	6 (7.4%)
	Other*	1 (1.2%)
Years providing primary care	< 5	25 (30.9%)
	5–9	13 (16.0%)
	10–19	18 (22.2%)
	> 20	25 (30.9%)
Received extra training in palliative care		43 (53.1%)

*n = 1 self-identified as social worker

### PCP perceptions of community-led ACP activities

The concept of ACP awareness and education workshops provided by trained volunteers within community-based non-profits in community settings was perceived to be potentially impactful by most respondents (82.7% responded moderate or greater impact; 67/81). Over 80% perceived a moderate or greater impact of access to community-led ACP education workshops which could be tailored for specific age or diagnosis groups (81.5%; 66/81) and educate the public about limitations of life-sustaining treatments (91.4%; 74/81) and informing patients and families about existing ACP activities and tools (83.8%; 68/81).

The most commonly endorsed reasons for not referring a patient to a community-led ACP activity were lack of awareness of the option in the local community (62.1%; 54/87), lack of awareness of the activities generally (44.8%; 39/87), not having the information handy (35.6%; 31/87) and not knowing how to refer a patient (29.9%; 26/87). Few respondents responded that ACP was not part of primary care (8.0%; 7/87), were concerned about how patients and family members would react (4.6%; 4/87) or did not see the value (2.3%; 2/87).

#### Desired outcomes of attendance at a community-led ACP activity

A total of 64 (74%; 64/87) respondents provided an answer when asked what they would like patients and family members to return to them knowing or having done after being referred to a community-led ACP activity. Five distinct categories emerged from the free text response analysis (Table 2).

**Table 2:** What PCPs want patients/family members to know or come back having done

Categories	Illustrative Quotes
Greater knowledge and understanding of ACP	“Clear understanding and awareness of ACP” “What some of the language around ACP (advance directives, no CPR etc.) means” “Further understanding about what various MOST [Medical orders for scope of treatment] statuses actually entail” “Knowledge of various means to sustain life in hospital and their limitations” “Learn about futile care and poor outcomes after resuscitation in many people” “Understanding scenarios that require certain types of decisions - i.e. that a tube feed can be temporary after a stroke, that dialysis can be temporary and can always be stopped after being started, and conditions under which SDMs [substitute decision maker] are called on.”
Reflections on personal wishes and self-empowerment	“Consider their values/what is most important to them, their goals of care, who their substitute decision maker might be” “Had some thoughtful assessment of their own situation” “Done some thinking about what quality of life they want for right now and what functional limitations would make life possibly not worth living in the future” “A feeling of empowerment over their health”
Discussions with family and close friends	“Had family discussions so everyone aware of patients desires and not come back to keep discussing family differences” “Explored […] their family’s emotions around ACP”
Readiness to continue discussion with GP	“Be willing to talk about a plan” “Feel heard + understood + safe to continue to conversation with me/team” “Have [their understanding of ACP] open the door for discussion with their physician” “Bringing back specific questions they have about their health or prognosis” “To ask primary care provider re medical conditions and prognosis/alternative treatment/care plans”
Paperwork and legal forms	“Know about […] legal affairs to get in order” “Legal forms/discussions to appoint SDM [substitute decision maker] and POA [power of attorney]” “Understanding of [Medical Orders for Scope of Treatment] forms” “A brief card with some of the decision points - come back with it”

Categories and illustrative quotes answering the question “what do you want patients/family members you refer to community-led advance care planning (ACP) activities to know or come back having done?”

#### Greater knowledge and understanding of ACP

PCPs reported wanting their patients to gain a greater understanding of ACP, including personal considerations to think about when engaging in ACP and knowledge of key terms and medical language such as *“advance directives, NO CPR etc.*”. PCPs also noted that patients should understand their options and know what specific medical orders entail. They highlighted the Medical Orders for Scope of Treatment (MOST) designations in BC as an example. In addition, emphasis on the importance of a realistic understanding of the limitations of treatment and the likelihood of outcomes emerged including *“knowledge of various means to sustain life in hospital and their limitations*”. Finally, respondents suggested the use of practical examples, such as common scenarios in treatment.

#### Reflections on personal wishes and self-empowerment

PCPs wanted their patients to have personally reflected on their values as well as wishes and preferences for care and to have at least thought about their goals of care and to have chosen a substitute decision maker. They also mentioned wanting patients to feel a sense of “*empowerment over their health*” and confidence in their care plan.

#### Discussions with family members and close friends

Respondents emphasized that the knowledge gained from community-led ACP activities should facilitate conversations between patients and their family and close friends regarding their values, goals and healthcare wishes. PCPs described wanting their patients to have “*explored their family’s emotions*” on ACP and shared their care preferences with in “*family discussions so everyone [is] aware of patients desires and [do] not come back to keep discussing family differences*”.

#### Readiness to continue discussion with PCP

Respondents expected that after attending a community-led ACP activity, patients should be willing to discuss their preferences, identify questions about their own health condition, and be prepared to create an ACP with their PCP. The hope was that they would “*Feel heard + understood + safe to continue to conversation with [their PCP]*”. Respondents also suggested that patients should know that specific questions about their own health and options would be clarified with their PCP.

#### Paperwork and legal forms

Respondents offered details on the documents they would like their patients to understand or to have completed after attending a community-led ACP activity. This included their documented preferences for care, which could include “*A brief card with some of the decision points*”, and “*Legal forms/discussions to appoint a Substitute Decision Maker (SDM) and Power of Attorney (PO*A)” as required. For example, participants noted the Medical Orders for Scope of Treatment forms and Representation Agreements, which are the relevant forms used in BC to assist with incapacity planning or to communicate medical orders arising from these discussions.

### Integrating primary care and community-led ACP activities

A total of 60 (69%; 60/87) respondents provided an answer regarding approaches to integrate primary care and community-led ACP activities. Five distinct categories emerged from the free text response analysis (Table 3).

**Table 3:** What PCPs think would be successful primary care and community-led ACP activity connection approaches

Categories	Illustrative Quotes
Public awareness and outreach	“Advertisement on news, in local paper, social media” “More public awareness of documents like care plans, MOST (Medical orders for scope of treatment), resuscitation orders” “Well clearly we need to know about them, but more importantly, they need to be out in the community, like assisted livings and senior centres, promoting discussion so they are not so surprised when I do.”
Hardcopy resources for patients	“Easy to read patient handouts/pamphlets” “Poster or handout that can be given to patient on how they can register for the session
Communication between activity coordinators and primary care providers	“Bringing the providers in both contexts together and have a discussion on how to best collaborate to provide patients with the conversations needed to provide ACP optimally” “Virtual meeting with primary care providers, or even just an email sent to the clinic for them to inform us 1. that they exist 2. how to refer 3. what services they provide” “Communication back to Primary care of attendance” “A summary of what was discussed at session to come to GP (general practitioner)”
Easy referral process	“Facilitator based in practices, perhaps connected through health authority” “Collaboration with primary care through patient care networks” “Through divisions of family practice” “Easy method of referral” “Easy to find form/info on Pathways” “Have a referral form available on all EMRs (electronic medical records)”
Follow-up for patient to connect with GP (general practitioner) after attending activity	“Have patients make an appointment with me once they’ve completed the activity” “Start conversation and direct to physician” “Form(s) to take to GP to complete the process”

Categories and illustrative quotes answering “what would be a successful approach to connect primary care and community-led advance care planning (ACP) activities?”

#### Public awareness and outreach

Respondents highlighted the importance of public awareness of community-led ACP activities. Strategies to raise awareness included advertising in social media and local papers and hosting the activities in community spaces, especially those for older adults such as libraries, seniors’ centres and assisted living homes. This was considered important “*so they are not so surprised*” when the PCP raises the topic.

#### Communication between ACP activity coordinators and PCPs

Respondents noted the need for communication between PCPs and community-led ACP activities, such as a virtual meeting or email. The communication could include information about the existence of the programs, what services they provide and referral information, and discussions about strategies for collaboration, such as updating PCPs when a patient of theirs has attended an ACP activity.

Leveraging the use of existing healthcare networks was suggested as a potential approach to connect PCPs and community-led ACP activities. In British Columbia, these include health authorities, primary care networks, and divisions of family practice.

#### Easy referral process

PCPs most frequently mentioned the importance of establishing an easy and accessible referral process to community-led activities, which could include the integration of referral forms into electronic medical records. Respondents also suggested adding information to referral web directories for physicians: “*…easy to find form/info on Pathways*”, which is a web-based directory for physicians in BC to access referral information. They also described the need for physical handouts for patients and posters that could be put up around their offices. The patient handouts would include information on ACP and community-led ACP activities such as how to register for the session.

#### Follow-up for patient to connect with PCP after attending activity

Respondents offered details on next steps for patients to follow-up with their PCPs after attending a community-led ACP activity. They suggested that *“a summary of what was discussed at session to come to GP”*, community-led activity coordinators should direct patients to “*make an appointment with [their PCP] once they’ve completed the activity*”, and that attendees should be provided with the appropriate ACP forms to fill out with their PCP.

## Discussion

In this survey of 87 PCPs, there was support expressed for community-led ACP initiatives among most respondents. The main reasons for not referring patients to these existing initiatives were lack of awareness of these activities and referral mechanisms. There was endorsement for efforts to increase awareness of community-led ACP initiatives and to create easy to use referral processes. PCPs articulated ways community-led ACP activities could prepare their patients for ACP discussions with their PCP and made specific recommendations for how they could link their patients to these initiatives.

Evidence is accumulating to support the notion that engaging patients in ACP outside the clinical context can influence healthcare provider behavior. Previous randomized trial evidence evaluating the online PREPARE tool designed to engage patients in ACP found that among patients who had used the tool, there was greater active patient participation in discussions with their PCP compared to the group who only completed an advance directive [28–30]. These findings are consistent with the perception among our respondents that a goal of community-led ACP was for patients to be better prepared to discuss ACP, and that community-led ACP could empower patients and encourage them to engage in a range of ACP elements. PCPs envisioned these elements to be undertaken outside the clinical context.

Despite the known barriers to implementing ACP in primary care that have been reported in the literature, PCPs perceived that they had a specific role in ACP. They saw their role as discussing the patient’s medical issues and answering the patient’s questions related to their health. These perceptions of how PCPs envision their role in ACP should be carefully considered in developing interventions for use in primary care, while also informing necessary PCP educational needs, otherwise barriers such as lack of time and perceived lack of patient readiness to engage may continue to undermine the success of interventions [31].

Undertaking ACP activities in the community, or ‘upstreaming’ ACP, into health promotion activities as part of a public health approach, has been identified as beneficial [21, 32, 33]. More than just saving healthcare providers’ time, this would aim to normalize these discussions and shift broader culture [32]. However, this requires a multi-pronged, cross-sector approach, [21, 33] of which community-led ACP activities can be an important piece. Group-based interventions can be as effective as individual discussions, [34] and lead to engagement in ACP behaviors such as document completion [35]. Groups have benefits over individual interventions, such as sharing of stories among the group, [21] with larger groups asking more questions and sharing more personal examples [36]. This requirement for a cross-sector approach aligns with our results indicating that initiatives should leverage PCPs’ existing networks such as the health authority and Divisions of Family Practice to share information, and patients should be prompted to contact their PCP for follow-up.

Primary care providers wished for their patients to learn about the limitations of some life-sustaining treatments and the scenarios where these decisions may be required, and they hoped their patients would consider and discuss their values around meaningful quality of life with their substitute decision maker. In the jurisdiction where this study was undertaken, the “medical orders for scope of treatment” form is used system-wide, which may explain respondents’ interest in discussing this medical order. These forms provide guidance around urgent healthcare decisions, and also provide guidance around the general types of treatment the patient should receive, aiming to put medical language around the person’s values and goals. There has been criticism of the focus of ACP being completion of advance directive documents and medical orders in advance of specific clinical decisions, [37, 38] with calls to shift the nature of conversations from specific healthcare decisions to understanding patients’ values and personal goals, and helping to align healthcare with these going forward [39, 40]. While the PCPs in our study indicated a desire for patients to know their preferences for a MOST designation after the community-led ACP initiatives, this would have been outside of the appropriate scope of our educational intervention. This may be signaling that PCPs may need some further education or support about translating conversations about patient goals and values into medical context and goals of treatments. Furthermore, the wish of PCPs for their patients to learn about specific treatments and clinical scenarios may not be a reasonable expectation from community-led ACP activities. It is important that those facilitating these activities stay within an established scope of practice, [41] which if it included information about specific treatments or clinical scenarios would be limited to provision of general information, akin to a decision aid [42]. Instead, the benefit of community-led ACP activities would be support in determining broader values and personal goals and priming participants for future decisions in the medical context. Application of these values and personal goals to an individual’s specific medical situation would likely require the support of a healthcare professional. Our results suggest that primary care providers see a role for themselves in these aspects of conversations.

### Strengths and limitations

There has been considerable research on interventions to increase ACP focusing on educating and preparing clinicians which do not overcome barriers to patient engagement and time constraint barriers. This study provides new insights on optimizing the clinician role through partnerships with community-based organizations and the use of trained peer facilitators to host and lead community-led ACP activities. A limitation of this study is that it was conducted in a single Canadian province and results may not be directly applicable to other jurisdictions. Response rates were lower than our target. As data collection took place in early 2021, we expect that this is due to the impact of the COVID-19 pandemic, with potential respondents likely under resultant high workloads, and were so less inclined to participate in the research study, although survey response rates for health professionals are typically low [43]. Additionally, utilizing existing mass communications with potential respondents rather than personalized invitations likely contributed to the lower response rate. Selection bias may have been present in that physicians who chose to respond may have been more interested in the topic of ACP than those who did not.

## Conclusions

In this sample of PCPs, community-led ACP activities were perceived to be useful to engage and prepare patients to continue ACP discussions with clinicians. Efforts should be made to establish and integrate community-led ACP initiatives within existing primary care systems to ensure awareness and uptake.

### Electronic supplementary material

Below is the link to the electronic supplementary material.

Supplementary Material 1

## Data Availability

The datasets used and/or analyzed during the current study are available from the corresponding author on reasonable request.

## Abbreviations

ACP: Advance Care Planning
BC: British Columbia
BCCPC: British Columbia Centre for Palliative Care
CPR: Cardio Pulmonary Resuscitation
EMR: Electronic Medical Record
GP: General Practitioner
MOST: Medical Orders for Scope of Treatment
PCP: Primary Care Provider
POA: Power of Attorney
SDM: Substitute Decision Maker
