# Supplementary material for: Primary care providers’ perceptions on the integration of community-led advance care planning activities with primary care: a cross-sectional survey
Source: BMC Prim Care. 2023 Sep 24;24:197. doi: 10.1186/s12875-023-02144-z (PMC10519084; doi:10.1186/s12875-023-02144-z)
Supplement: Supplementary file 1 — Supplementary Material 1 [file 12875_2023_2144_MOESM1_ESM.docx]

**[BEGINNING OF SURVEY]**

# Advance Care Planning (ACP) in Primary Care

In this first section of the survey, we would like to find out about **your** ACP engagement.

#### In your opinion, how important is it that primary care providers have Advance Care Planning (ACP) conversations with their patients?

| **Not important at all** | | **Slightly important** | **Moderately important** | **Very important** | **Extremely important** | |
| --- | --- | --- | --- | --- | --- | --- |
| 1 | 2 | | 3 | 4 | | 5 |

#### How often do you talk to your patients 50 years and older about issues related to ACP?

|  | **Never** | **Less than once a month** | **At least once a month** | **At least once a week** | **Several times a week** | **(Almost) Every day** |
| --- | --- | --- | --- | --- | --- | --- |
|  | 0 | 1 | 2 | 3 | 4 | 5 |
| Prior to the COVID-19 Pandemic |  |  |  |  |  |  |
| Since the COVID-19 Pandemic |  |  |  |  |  |  |

*(If question #2 is answered with “never” twice, skip to question #8.)*

#### Who typically initiates ACP discussions?

|  | **Always myself/ a colleague** | | **More often myself/ a colleague** | | **About equal myself/ a colleague and patient or their family** | **More often the patient or their family** | **Always the patient or their family** |
| --- | --- | --- | --- | --- | --- | --- | --- |
|  | **0** | **1** | | **2** | | **3** | **4** |
| Prior to the COVID-19 Pandemic |  | |  | |  |  |  |
| Since the COVID-19 Pandemic |  | |  | |  |  |  |

#### In your opinion, when is the most appropriate time to have ACP discussions with your patients? (select all that apply)

| When a patient asks about it |  |
| --- | --- |
| Unplanned hospitalization or emergency visit |  |
| Change in health status or trajectory of illness |  |
| Diagnosis of a serious illness |  |
| When a patient reaches a particular age |  |
| At an annual health exam visit |  |
| Other (please specify): __________________________________________________ |  |

#### When having ACP discussions with your patients, how often do you discuss the following?

|  | **Never** | **Rarely** | **Sometimes** | **Often** | **Always** |
| --- | --- | --- | --- | --- | --- |
|  | 0 | 1 | 2 | 3 | 4 |
| Patient’s understanding of their health condition or illness |  |  |  |  |  |
| Patient’s prognosis and the expected outcomes of treatment |  |  |  |  |  |
| Patient’s values, wishes, and worries relating to their future healthcare |  |  |  |  |  |
| Who can speak for the patient if they can’t speak for themselves *(e.g., substitute decision maker)* |  |  |  |  |  |
| The role of life-support interventions and life-sustaining measures, and their expected degree of benefit |  |  |  |  |  |
| Official documents *(e.g., No-CPR/DNR Form, Medical Order for Scope of Treatment, Advance Directives)* |  |  |  |  |  |
| Other resources *(e.g., palliative care, social work,  spiritual care)* |  |  |  |  |  |
| Other (please specify): ___________________ |  |  |  |  |  |

#### How would you rate your skills in having ACP discussions with your patients?

| **Limited** | **Fair** | **Average**  (comparable to colleagues) | **Very good** | **Expert** |
| --- | --- | --- | --- | --- |
| 1 | 2 | 3 | 4 | 5 |

#### Which of the following resources have you used and found helpful to discuss ACP with your patients?

|  | **Used and helpful** | **Used but unhelpful** | **Never used, but aware of it** | **Unfamiliar to me** |
| --- | --- | --- | --- | --- |
|  | 1 | 2 | 3 | 4 |
| Serious Illness Conversation Guide (SICG) |  |  |  |  |
| Speak Up: ACP Quick Guide & workbook |  |  |  |  |
| My Voice: Expressing My wishes for Future Health Care Treatment (Guide) |  |  |  |  |
| Conversation starters i.e. “Just Ask” – conversation card and booklet |  |  |  |  |
| Resources provided by lawyers, notaries, legal organizations |  |  |  |  |
| Health authority resources (please specify): ______________________________________ |  |  |  |  |
| Other (please specify): ______________________________________ |  |  |  |  |

#### Which barriers have you encountered that prevent you from having ACP discussions with your patients?

Barriers related to **your own role**: _________________________________________________________

Barriers related to **healthcare system or external factors**: ______________________________________

Barriers related to **patient’s attitudes or characteristics**: _______________________________________

#### In your opinion, which of the following measures aiming to make it easier for you to discuss ACP with your patients would have the most impact?

Implementation would be by health authorities or other applicable organizations.

|  | **No impact** | **Minor impact** | | **Moderate impact** | | **Major impact** | | **Severe impact** |
| --- | --- | --- | --- | --- | --- | --- | --- | --- |
|  | 0 | 1 | | 2 | | 3 | | 4 |
| **Public engagement & awareness** |  |  | |  | |  | |  |
| Providing ACP education/workshops in community settings **for the public** |  |  | |  | |  | |  |
| Targeted ACP education/workshops in community settings **for specific (age/diagnoses) groups** |  |  | |  | |  | |  |
| Educating the public about limitations of life-sustaining treatments |  |  | |  | |  | |  |
| Informing patients and families about existing ACP activities and tools |  |  | |  | |  | |  |
| **Enhancing capacity for ACP in primary care** |  |  | |  | |  | |  |
| Clarifying the role of different primary care providers in ACP |  |  | |  | |  | |  |
| Training for primary care providers in ACP/Serious Illness Conversations |  |  | |  | |  | |  |
| **Integration of ACP into the family medicine clinic workflow** |  |  | |  | |  | |  |
| Improving access to ACP resources |  |  | |  | |  | |  |
| Implementing a mechanism to document ACP |  |  | |  | |  | |  |
| Implementing automated reminder mechanisms for ACP within EMR |  |  | |  | |  | |  |
| **Increasing system and policy support** |  |  | |  | |  | |  |
| Providing/increasing transferability of ACP between healthcare settings |  |  | |  | |  | |  |
| Providing/increasing remuneration for ACP |  |  | |  | |  | |  |
| Providing/increasing clinical pathways and guidelines for ACP in primary care |  |  | |  | |  | |  |
| **Other measures** |  |  | |  | |  | |  |
| Please specify: ________________________ |  | |  | |  |  |  | |

# Facilitating Advance Care Planning through community-based approaches

Over the past several years, the BC Centre for Palliative Care has been training community-based organizations to deliver ACP education sessions and activities. The evidence suggests that these sessions are very successful in engaging the public and preparing them for conversations with their health care team.

The following questions focus on the ways that partnerships/connections between primary care and community-based approaches can support or facilitate ACP done by primary care providers.

#### Are you aware of any community-based organizations offering education for the public on ACP?

| Yes | No | If “Yes”, please specify: _______________________________________ |
| --- | --- | --- |

#### Have you ever referred a patient to a community-led ACP activity such as a conversation game or an ACP education workshop?

| Yes | No |
| --- | --- |

*(If “no”)* **Please explain why not** (select all that apply):

| I didn’t think of it/not part of primary care. |  |
| --- | --- |
| I don’t discuss ACP with my patients. |  |
| I wouldn’t know how to refer a patient to community-led ACP activities. |  |
| I am not aware of any community-led ACP activities in my local community. |  |
| I am not aware of the existence of community-led ACP activities in general. |  |
| I don’t see the value/benefit of referring my patients to community-led ACP activities. |  |
| I don’t have the information about community-led ACP activities handy. |  |
| I’m concerned about how patients/families will react to the suggestion. |  |
| There are too many barriers to referring patients *(e.g., lack of time in discussing referral with patients, lack of information about such activities).* |  |
| Other (please specify): _______________________________________________ |  |

*(If “yes”)* **What was your reason for referring your patient(s)?** (select all that apply)

| Other patients found it helpful. |  |
| --- | --- |
| Family members of other patients found it helpful/helped other family members understand ACP options. |  |
| Patients who attended a community-led ACP activity were more knowledgeable about ACP/open to discuss ACP. |  |
| Other (please specify): _______________________________________________ |  |

#### Based on current evidence, below are some statements about community-led ACP activities. Please indicate your level of agreement with the following statements:

|  | **Strongly disagree** | **Somewhat disagree** | **Neither agree nor disagree** | **Somewhat agree** | **Strongly agree** |
| --- | --- | --- | --- | --- | --- |
|  | 0 | 1 | 2 | 3 | 4 |
| **Community-led ACP activities…** |  |  |  |  |  |
| …promote public awareness. |  |  |  |  |  |
| …promote greater level of ACP knowledge among patients. |  |  |  |  |  |
| …break down barriers/make it easier for me to have to ACP discussions with patients. |  |  |  |  |  |
| …are helpful for the patient. |  |  |  |  |  |
| …are helpful for the patient’s family. |  |  |  |  |  |

#### If available in your community/area in the future, how likely is it that you will refer patients to community-led ACP activities?

| Extremely unlikely | | Somewhat unlikely | Neither likely nor unlikely | Somewhat likely | Extremely likely | |
| --- | --- | --- | --- | --- | --- | --- |
| 1 | 2 | | 3 | 4 | | 5 |

*(If “extremely or somewhat unlikely”)* **Please explain why it is unlikely that you will refer patients to community-led ACP activities in the future** (select all that apply):

| I don’t discuss ACP with my patients. |  |
| --- | --- |
| I wouldn’t know how to refer a patient to community-led ACP activities. |  |
| I am not aware of any community-led ACP activities in my local community. |  |
| I am not aware of the existence of community-led ACP activities in general. |  |
| I don’t see the value/benefit of referring my patients to community-led ACP activities. |  |
| I don’t have the information about community-led ACP activities handy. |  |
| I’m concerned about how patients/families will react to the suggestion. |  |
| There are too many barriers to referring patients *(e.g., lack of time in discussing referral with patients, lack of information about such activities)*. |  |
| Other (please specify): _______________________________________________ |  |

*(If “neither")* **Why/why not?** ______________________________________________________

*(If “somewhat or extremely likely”)* **What do you want patients/family members you refer to community-led ACP activities to know or come back having done?** ________________________________________________________________________

#### In your opinion, what would be a successful approach to connect primary care and community-led ACP activities?

______________________________________________________________________________

# Demographics

#### What gender do you identify with?

| Male | Female | Other | Prefer not to disclose |
| --- | --- | --- | --- |

#### What is your age?

| Less than 35 years old |  |
| --- | --- |
| 35-44 years old |  |
| 45-54 years old |  |
| 55-64 years old |  |
| 65-74 years old |  |
| 75 years or older |  |

#### What kind of primary care provider are you?

| Family physician/general practitioner |  |
| --- | --- |
| Nurse practitioner |  |
| Other (please specify): __________ |  |

#### How many years have you provided primary care?

| Less than 5 years |  |
| --- | --- |
| 5-9 years |  |
| 10-19 years |  |
| 20 years or more |  |

#### Which best describes the area you practice in?

| Rural | Urban | Other |
| --- | --- | --- |

#### In which health authority is your practice located?

| Vancouver Coastal region |  |
| --- | --- |
| Fraser Health region |  |
| Vancouver Island |  |
| Interior region |  |
| Northern Health |  |
| Other (please specify): _______________ |  |

#### Which of the following best describes your practice? (select all that apply)

| Solo practice |  |
| --- | --- |
| Group practice (# of practitioners in the practice: ____________________) |  |
| Walk-in clinic |  |
| Long term care facilities |  |
| Primary care group model (primary care networks, family health team) |  |
| Practice includes other professionals *(e.g., Nursing, social work, pharmacy, OT/PT)* |  |
| Specialty/focused practice (please specify): _______________________________ |  |
| Other (please specify): _______________________________________________ |  |

#### What is your estimated practice size (# of patients per year)?

| Less than 1,000 patients |  |
| --- | --- |
| 1,000-1,999 patients |  |
| More than 2,000 patients |  |

#### Have you had any additional education, training or certification in End-of-Life Care, Palliative Care, or Serious Illness conversations?

| Yes | No |
| --- | --- |

*(If “yes”)* Please describe type of education, training or certification received: _______________________________________________________________

# Thank you for participating in this survey!

Please click HERE to provide your contact information to **choose your gift card** (available to the first 150 respondents) and indicate whether you would be interested in learning more about community-led ACP interventions, or participate in a potential future research study looking at the integration of community-led ACP interventions with primary care, or to learn about other activities being conducted by the BC Centre for Palliative Care.

The above link will take you to a separate survey not associated with the responses provided in this survey, and we will be unable to link your survey answers to your contact information.

**[END OF SURVEY]**
